# Large-Scale Gastric Cancer Susceptibility Gene Identification Based on Gradient Boosting Decision Tree

**DOI:** 10.3389/fmolb.2021.815243

**Published:** 2022-01-13

**Authors:** Qing Chen, Ji Zhang, Banghe Bao, Fan Zhang, Jie Zhou

**Affiliations:** ^1^ Department of Hepatobiliary Surgery, Union Hospital, Tongji Medical College, Huazhong University of Science and Technology, Wuhan, China; ^2^ Department of Pathology, Union Hospital, Tongji Medical College, Huazhong University of Science and Technology, Wuhan, China; ^3^ Wuhan Asia General Hospital, Wuhan, China; ^4^ Department of Biochemistry and Molecular Biology, Tongji Medical College, Huazhong University of Science and Technology, Wuhan, China

**Keywords:** gastric cancer, susceptibility gene, gradient boosting decision tree (GBDT), random walk (RW), gastric cancer-related genes

## Abstract

The early clinical symptoms of gastric cancer are not obvious, and metastasis may have occurred at the time of treatment. Poor prognosis is one of the important reasons for the high mortality of gastric cancer. Therefore, the identification of gastric cancer-related genes can be used as relevant markers for diagnosis and treatment to improve diagnosis precision and guide personalized treatment. In order to further reveal the pathogenesis of gastric cancer at the gene level, we proposed a method based on Gradient Boosting Decision Tree (GBDT) to identify the susceptible genes of gastric cancer through gene interaction network. Based on the known genes related to gastric cancer, we collected more genes which can interact with them and constructed a gene interaction network. Random Walk was used to extract network association of each gene and we used GBDT to identify the gastric cancer-related genes. To verify the AUC and AUPR of our algorithm, we implemented 10-fold cross-validation. GBDT achieved AUC as 0.89 and AUPR as 0.81. We selected four other methods to compare with GBDT and found GBDT performed best.

## Introduction

There are about 950,000 new cases of gastric cancer worldwide each year, and nearly 700,000 deaths. It is one of the most serious tumors (Rawla and Barsouk, 2019). The early clinical symptoms of gastric cancer are not obvious, and metastasis may have occurred at the time of treatment (Axon, 2006). Poor prognosis is one of the important reasons for the high mortality of gastric cancer (Eguchi et al., 2003). Therefore, the identification of gastric cancer-related genes can be used as relevant markers for diagnosis and treatment to improve diagnosis precision and guide personalized treatment (Duffy et al., 2014).

Identifying gastric cancer-related genes plays an important role in the treatment of gastric cancer. Research on metastasis-related genes is conducive to timely detection of early metastasis, screening of new markers and therapeutic targets, thereby improving the survival rate of patients (Arturi et al., 1997). Using animal models to screen gastric cancer metastasis-related genes (Wang and Chen, 2002), fully mimic the process of tumor metastasis *in vivo*, with high metastasis efficiency, clear phenotypic characteristics, and good clinical similarity. Cell line derived xenograft (CDX) model is a tumor model constructed by transplanting cultured tumor cells into immunodeficient mice (Georges et al., 2019). The cell lines used in the CDX model have been cultured *in vitro* for many generations, and their biological characteristics have changed significantly. Some tumor cell lines that adapt to culture *in vitro* and have metastatic potential have been selected, so it is easy to obtain the metastasis model. The establishment of the CDX model can be realized by subcutaneous injection, intraperitoneal injection, caudal vein injection, and so on (Lallo et al., 2017). Zhu et al. (2020) established a xenotransplantation model by subcutaneous injection of gastric cancer cell line BGC-823 into the hind limbs of nude mice. They found that mir-106a had the potential to promote tumor growth by targeting Smad7. At the same time, they found that mir-106a was related to peritoneal metastasis of gastric cancer. At present, studies have found that gastrin level has a strong relationship with the development of gastric cancer. Zu et al. (2018) successfully established a cell xenotransplantation model by subcutaneous injection of human gastric cancer cell line SGC-7901 in nude mice. They found that gastrin can inhibit the proliferation of poorly differentiated gastric cancer cells and enhance the inhibitory effect of cisplatin on gastric cancer by activating erk-p65-mir 23a/27a/24 axis. Tumor cells with biological enzyme markers can also be used to establish a CDX model (Agashe and Kurzrock, 2020), which is helpful to dynamically monitor tumor metastasis *in vivo* and facilitate the screening of metastasis related genes. Miwa et al. (2019) successfully established the intraperitoneal metastasis model by injecting MKN1 (MKN1 LUC) and MKN45 (MKN45 LUC) gastric cancer cells stably expressing luciferase and n87, Kato III, nugc4, and ocum-1 gastric cancer cells into the abdominal cavity of nude mice. The liver metastasis model was successfully established by injecting MKN1 Luc and MKN45 Luc directly into the portal vein of mice. Because the establishment of CDX model uses passage cell lines and lacks the microenvironment of tumor growth in human body (Lallo et al., 2017), it cannot well simulate the growth and metastasis of tumor in the human body. Patient derived cell models (PDC) use patient derived tumor cells isolated from malignant effusions such as ascites and pleural effusion (Bolck et al., 2019). Therefore, it can better reflect the individualized characteristics of patients and show unique advantages in the screening of tumor metastasis related genes and clinical drug screening. Lee et al. (2015) established a PDC model with cells collected from patients with metastatic cancer. The study found that the genomic changes of primary tumor and offspring PDC model were highly consistent, and the correlation of average variant allele frequency was 0.878. Further compared the genomic characteristics of primary tumor P0, P1, and P2 cells, and found that three samples (P0, P1, and P2 cells) were highly correlated. The drug response of the model reflects the clinical response of patients to targeted drugs. Although the PDC model established by metastatic patient derived tumor cells can reflect the individualized characteristics of patients, it is cultured *in vitro*, which is difficult to culture and cannot simulate the process of tumor metastasis *in vivo*. Therefore, the use of this model to screen metastasis related genes is limited. The metastasis related genes screened by the above CDX model and PDC model are conducive to the discovery of relevant molecules promoting gastric cancer metastasis and provide help for the early detection of gastric cancer metastasis in the clinic (Almagro et al., 2014). Patient derived xenograft (PDX) model improves the shortcomings of the CDX model and the PDC model. It is a better model to screen metastasis related genes at present. The model is a xenotransplantation model established by transplanting fresh clinical surgical specimens into immunodeficient mice. It maintains the microenvironment of primary tumor growth, so it can better simulate the biological behavior of tumors *in vivo*. Choi et al. (2016) successfully established 15 cases of gastric cancer PDX models, and found that the histological and genetic characteristics of the tumor models remained stable in subsequent passages and were highly consistent with the primary tumor. This discovery made the use of PDX models for the development of gastric cancer molecules possible. Research and individualized treatment are possible. The PDX model has relatively consistent genomics characteristics with the primary tumor, which is very conducive to the screening of individualized metastasis-related genes. Zhang et al. (2015) successfully established 32 PDX models of gastric cancer, and found that the gene amplification of FGFR2, MET, and ERBB2 is very similar between PDX models and their parent tumors, and the expression of PTEN and MET proteins are also moderately consistent. These data are *in vivo* testing of individualized therapy and screening of transfer-related genes provides a theoretical basis. There are many methods of tissue transplantation when establishing a PDX model, including subcutaneous transplantation, renal capsule transplantation, orthotopic transplantation, etc. (Okada et al., 2018). Among them, subcutaneous transplantation is the most commonly used transplantation method. Guo et al. (2019) established a PDX model of gastric cancer by subcutaneous transplantation and revealed the molecular mechanism of ISL1 that promotes gastric cancer metastasis by combining the ZEB1 promoter and the cofactor SETD7. ISL1 may be a potential prognostic marker of gastric cancer. Because the microenvironment of orthotopic transplantation tumors is closer to the human environment, orthotopic transplantation can simulate the growth of tumors in the human body better than subcutaneous transplantation, and it is easier to simulate clinical metastasis, which is beneficial to screening metastasis-related genes. Wang et al. (2018) found that 28 miRNAs are differentially expressed in invasive gastric cancer through array analysis. Among these 28 miRNAs, miR-29b is one of the most significantly down-regulated miRNAs. RNA response element (miRNA response element, MRE) binds to the negative regulation of MMP2, thereby affecting the development of gastric cancer.

However, this kind of animal model experiment method is very costly and time consuming. With the continuous enhancement of computing power, computing methods have been able to process massive amounts of biological data and mine knowledge from the data (Zhao et al., 2021). Deep learning, machine learning, and reinforcement learning have been widely used in the fields of biology and medicine (Zhao et al., 2020a; Tianyi et al., 2020). These methods use existing knowledge to construct complex mathematical models to predict new knowledge (Zhao et al., 2020b). In this paper, we extracted network association of each gene by Random Walk (RW) and used GBDT to identify the gastric cancer-related genes.

## Method

We obtained 435 genes that are known to be related to gastric cancer in DisGeNet (Piñero et al., 2020). We collected genes that can interact with these 896 genes in HumanNet V2.0 (Hwang et al., 2019). Based on the interaction information, we built a gene interaction network. This network contains 1331 nodes, and each node is a gene.

### Extracting Features by RW

The core formula of RW is as follows:

$$P_{t+1}=\left(1-\gamma \right)AP_{t}+\gamma P_{0}$$ (1)

A is the adjacency matrix of the gene interaction network. P is random walk matrix. γ is a parameter that is needed to be set. We set γ as 0.5 based on experience.

If $\left\|P_{t+1}-P_{t}\right\|>\ell$ (we can set ℓ as arbitrarily small number), we can repeat Formula (1). Otherwise, we could obtain $P_{t+1}$ as the final RW matrix.

### Identifying Gastric Cancer Susceptibility Gene by GBDT

After obtaining the feature of genes by RW, we need to build a classifier to identify whether a gene is associated with gastric cancer GBDT does not need to scale the data to build model, and it is also suitable for data sets where dual features and continuous features exist at the same time. First, the decision tree used by GBDT is a CART regression tree. Whether it is dealing with regression problems or two classifications and multiple classifications, the decision trees used by GBDT are all CART regression trees. Because the gradient value to be fitted in each iteration of GBDT is a continuous value, a regression tree is used. The most important thing for the regression tree algorithm is to find the best division point, then the division point in the regression tree contains all the desirable values of all features. The criterion for the best division point in the classification tree is entropy or Gini coefficient, which are both measured by purity, but the sample labels in the regression tree are continuous values, so it is no longer appropriate to use indicators such as entropy, instead of the square error, which can judge the degree of fit very well.

The process of constructing CART is as follows:

Input: training data set D. Output: regression tree f (x).

Recursively divide each region into two sub-regions in the input space where the training data set is located and determine the output value on each sub-region to construct a binary decision tree:

$$\min\left[\min{\sum \left(y_{i}-c_{1}\right)}^{2}+\min{\sum \left(y_{i}-c_{2}\right)}^{2}\right]$$ (2)

As shown in Formula (2), we need to choose (j, s) to minimize $\min{\sum \left(y_{i}-c_{1}\right)}^{2}+\min{\sum \left(y_{i}-c_{2}\right)}^{2}$ . Then, we need to introduce (j, s) to divide the area and determine the corresponding output value:

R1(j,s)=x|x(j)≤s,R2(j,s)=x|x(j)>s (3)

$${\overset{⌢}{c}}_{m}=\frac{1}{N}\sum_{x1\in R_{m}\left(j,s\right)}y_{i},x\in R_{m},m=1,2$$ (4)

Continue to call Steps (1) and (2) for the two sub-regions until the stop condition is met.

Divide the input space into M regions $\left(R_{1},R_{2},...,R_{m}\right)$ , build the final decision tree.

$$f\left(x\right)=\sum_{m=1}^{M}{\overset{⌢}{c}}_{m}I\left(x\in R_{m}\right)$$ (5)

Gradient boosting is an improved algorithm of the Boosting Tree. There are three steps to implement the Boosting Tree.

Step 1 Initialize $f_{0}\left(x\right)=0$ .

Step 2 Calculate residual $r_{mi}=y_{i}-f_{m-1}\left(x\right),i=1,2,\ldots ,N$ .

Step 3 Fit the residual $r_{mi}$ to obtain regression tree and obtain $h_{m}\left(x\right)$ .

Step 4 Update $f_{m}\left(x\right)$ , $f_{m}\left(x\right)=f_{m-1}\left(x\right)+h_{m}\left(x\right)$ .

Step 5 The final regression boosting tree would be: $f_{M}\left(x\right)=\sum_{m=1}^{M}h_{m}\left(x\right)$ .

Based on the Decision Tree and Gradient Boosting, we can combine them to obtain the final GBDT.

First, we need to initialize week learner.

$$f_{0}\left(x\right)=\mathrm{argmi}n_{c}\sum_{i=1}^{N}L\left(y_{i},c\right)$$ (6)

For each sample i = 1, 2,..., N, we need to calculate the negative gradient (residual):

$$r_{im}=-{\left[\frac{\partial L\left(y_{i},f\left(x_{i}\right)\right)}{\partial f\left(x_{i}\right)}\right]}_{f\left(x\right)=f_{m-1}\left(x\right)}$$ (7)

Use the residual obtained in the previous step as the new true value of the sample and use $\left(x_{i},r_{im}\right)$ as the training data of the next tree to obtain the new regression tree $f_{m}\left(x\right)$ . The leaf node area of $f_{m}\left(x\right)$ is $R_{jm},j=1,2,\ldots ,J$ . J is the number of leaf nodes.

### Calculate the Best Fit Value

$$\gamma_{jm}=\mathrm{argmin}\sum_{x_{i}\in R_{jm}}L\left(y_{i},f_{m-1}\left(x_{i}\right)+\gamma \right)$$ (8)

### Update Strong Learner

$$f_{m}\left(x\right)=f_{m-1}\left(x\right)+\sum_{j=1}^{J}\gamma_{jm}I\left(x\in R_{jm}\right)$$ (9)

### Get the Final Learner

$$f\left(x\right)=f_{M}\left(x\right)=f_{0}\left(x\right)+\sum_{m=1}^{M}\sum_{j=1}^{J}\gamma_{jm}I\left(x\in R_{jm}\right)$$ (10)

## Results

Since we obtained 435 genes that are known to be related to gastric cancer in DisGeNet and 896 genes that have strong interaction with them, the 435 genes were used as the positive samples and 896 were used as negative samples. We used these data to build GBDT model to identify gastric cancer susceptibility genes.

**FIGURE 1:**
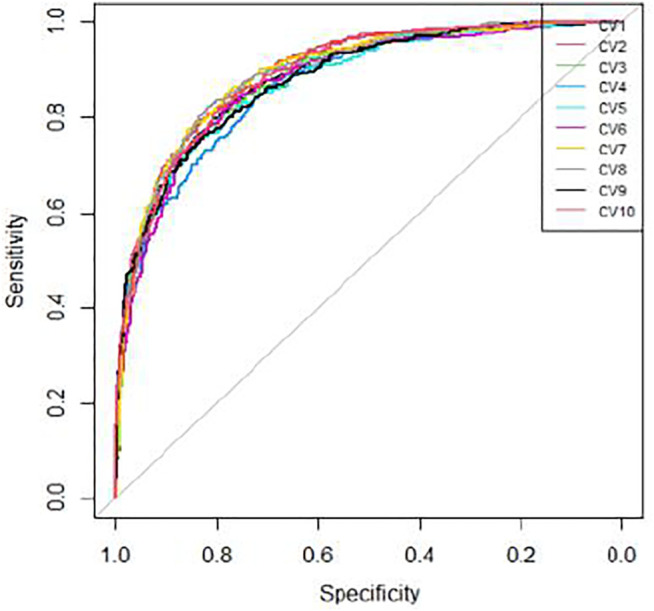
ROC curves of 10-cross validation.

**FIGURE 2:**
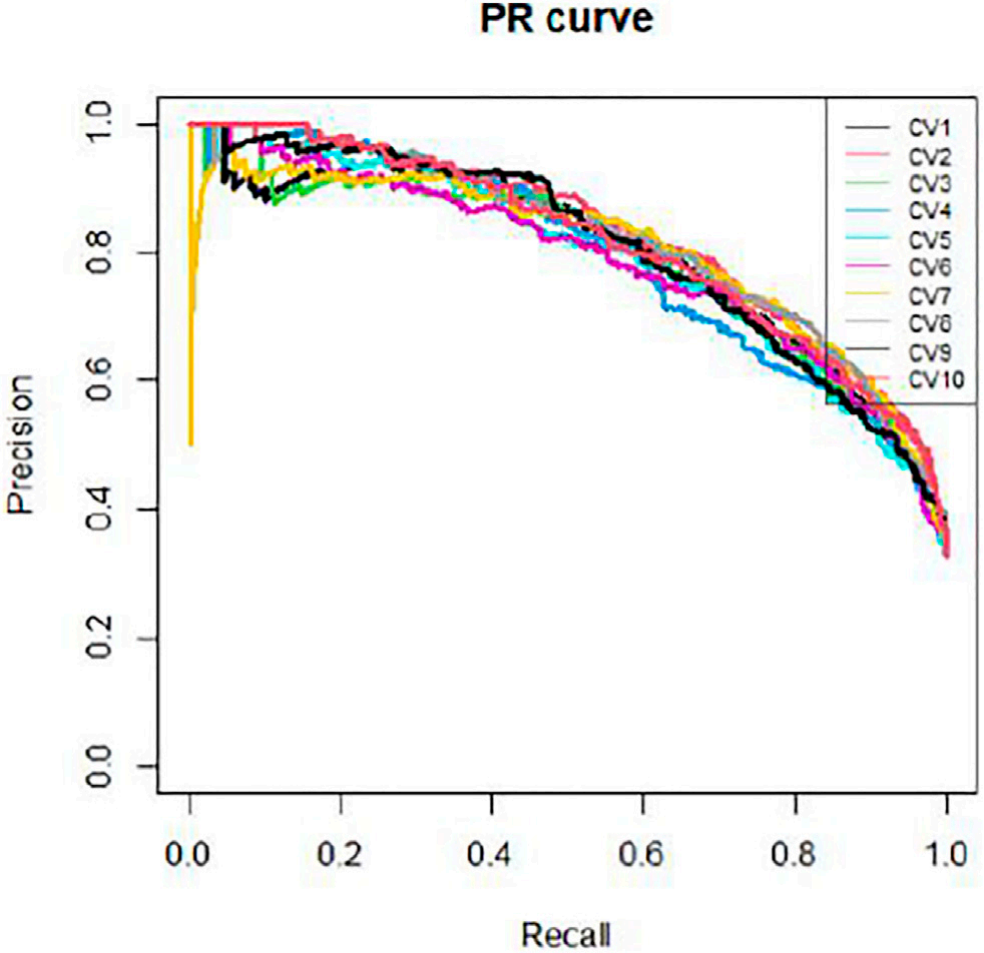
PR curves of 10-cross validation.

**FIGURE 3:**
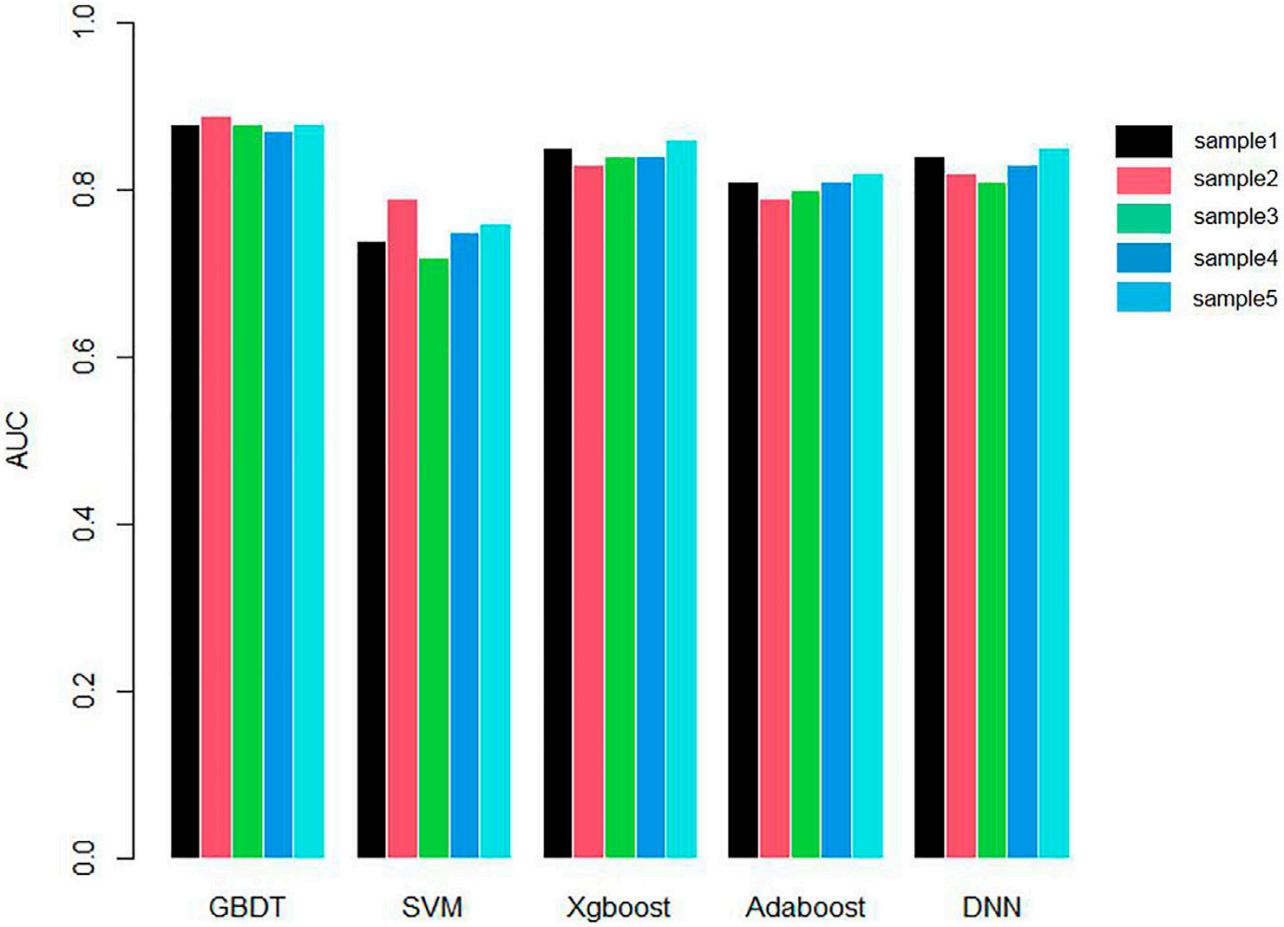
Comparison chart of AUC values of five methods.

**FIGURE 4:**
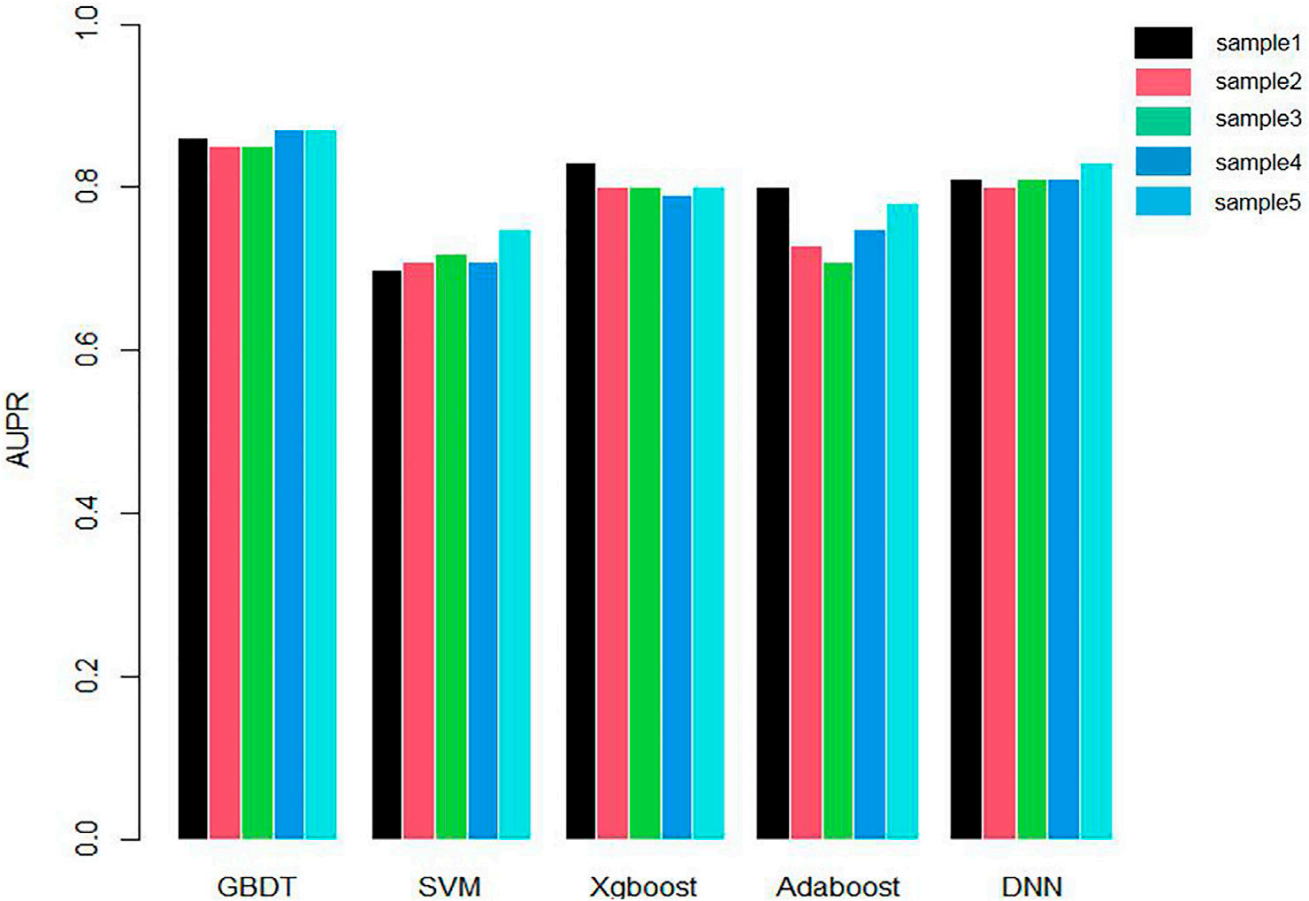
Comparison chart of AUPR values of five methods.

We applied 10-cross validation to verify the accuracy of our model. The AUC (Area Under Curve) and AUPR (Area Under Precision Curve) of our model is shown as Figures 1 and 2, respectively. The average AUC of 10-cross validation is 0.89 ± 0.008 and average AUPR of 10-cross validation is 0.81 ± 0.006. Since the number of negative samples is significantly higher than positive samples, to balance the training sample set, we randomly selected 435 negative samples from 896 genes each time and repeat the 10-cross validation. In addition, we also compared our method with other methods, such as Support Vector Machine (SVM), Xgboost, Adaboost, and Deep Neural Network (DNN). We totally randomly sampled five negative sets. The performance of these methods is shown as Figures 3 and 4.

As shown in Figures 3 and 4, the AUC and AUPR of GBDT are higher than other methods, which explains the superiority of our method over other methods.

## Conclusion

Through early detection, early diagnosis, and early treatment, the cure rate of patients with early gastric cancer can reach 85%; However, the 5-year survival rate of patients with advanced gastric cancer is less than 10%. At present, inhibitors targeting vascular endothelial growth factor (VEGF), epidermal growth factor (EGF), and tyrosine kinase have been successfully developed, showing significant curative effects on gastric cancer. This greatly encourages us to study the characteristic markers of recurrence or metastasis of gastric cancer from the perspective of genes. Few genes related to gastric cancer have been found in cohort studies and animal model experiments. However, due to the cost, such methods cannot be popularized large scale.

In this paper, we proposed a novel method to identify gastric cancer-related genes in large scale. Genes that interact more closely are more likely to be related to similar diseases. Based on this hypothesis, we considered to use the gene interaction information to build a network and infer the gastric cancer-related genes by this network. RW was applied to encode the features of genes and GBDT was implemented to identify gastric cancer-related genes. We verified our method by two kinds of 10-cross validation experiments. Our method showed high accuracy in both experiments, indicating that our method can be used to identify genes related to liver fibrosis. The method proposed in this article will provide guidance for genetic mechanism and clinical treatment of gastric cancer.

## Data Availability

The datasets presented in this study can be found in online repositories. The names of the repository/repositories and accession number(s) can be found in the article/Supplementary Material.
